# Quantifying Older Black Americans’ Exposure to Structural Racial Discrimination: How Can We Measure the Water In Which We Swim?

**DOI:** 10.1007/s11524-022-00626-6

**Published:** 2022-04-29

**Authors:** S. E. LaFave, K. Bandeen-Roche, G. Gee, R. J. Thorpe, Q. Li, D. Crews, L. Samuel, A. Cooke, M. Hladek, Sarah L. Szanton

**Affiliations:** 1 Johns Hopkins School of Nursing, Baltimore, MD USA; 2 Johns Hopkins Bloomberg School of Public Health, Baltimore, MD USA; 3 UCLA Fielding School of Public Health, Los Angeles, CA USA; 4 Johns Hopkins School of Medicine, Baltimore, MD USA

**Keywords:** Racism, Discrimination, Lifecourse, Measurement

## Abstract

**Abstract:**

The USA was built on legalized racism that started with enslavement and continues in the form of structural racial discrimination. This discrimination is difficult to measure because its many manifestations are hard to observe and dynamic. A useful tool would measure across settings, institutions, time periods in a person’s life and the country’s history. The purpose of this study was to design a measure of structural racial discrimination that meets those criteria and can be used in large national datasets. To do this, we started with an exploratory mixed-methods instrument design, including qualitative interviews with 15 older Black adults and focus groups with 38 discrimination researchers and other key stakeholders. We then identified 27 indicators of structural racial discrimination across nine theorized discrimination contexts. We matched these with historical administrative data sets to develop an instrument that could quantify older Black Americans’ exposure to structural racial discrimination across contexts, the life course, and geographies. These can be mapped to the life course of structural discrimination based on the home addresses of those surveyed. Linking these to available indicators is a promising approach. It is a low burden for participants and enables increasingly multifaceted and focused measurement as more national datasets become available. A flexible, feasible comprehensive measure of structural discrimination could allow not only more thorough documentation of inequities but also allow informed decision making about policies and programs intended to promote racial equity.

**Significance Statement:**

To our knowledge, this is the first study that presents a framework for assessing structural racial discrimination across contexts, life course, and geography that is grounded in theory and in the lived experience of intended participants. Leading researchers and policy makers have called for improved measures of structural racism and discrimination and specifically for a lifecourse approach to measurement. This study is a step in that direction.

**Classification:**

Social Sciences

## Introduction

The US economy was built on a foundation of people who were enslaved. Since emancipation, an explicit and implicit hierarchy that values white over black and brown human life has been implemented through racial discrimination. Structural discrimination is “the totality of ways in which societies foster racial discrimination through mutually reinforcing systems including housing, education, employment, earnings, civil representation, benefits, credit, media, health care, and criminal justice. These patterns and practices in turn reinforce discriminatory beliefs, values, and distribution of resources.” [1] These patterns and practices affect a person across contexts and throughout the life course. It is multifaceted, ubiquitous, and dynamic—it’s not “the shark” but rather the water each American swims in [2].

While structural discrimination is important because it is so pervasive and detrimental, measuring it is difficult. To date, researchers have relied primarily on self-report to understand the impact of discrimination. As examples, the Everyday Discrimination Scale [3] and Major Experiences of Discrimination Scale [4] ask participants about the types and frequency of discrimination experienced, either in their day-to-day interactions with others (interpersonal discrimination) [5] or within specific institutions such as a school or workplace (institutional discrimination) [5]. Self-report of discrimination requires individuals to assess whether or not they are being treated unfairly (e.g., is this store clerk disrespectful to all customers, or is he particularly disrespectful to me?) [6]. It therefore has the limitation that individuals may lack the information needed to assess their own exposure to discrimination, particularly at the structural level. For example, a worker may not know of the salaries of their peers and may not be aware that their employer pays racial/ethnic minority workers less than white workers. Self-report of discrimination, moreover, has been associated with anxiety and depression, [7] hypertension [8], and obesity [9]. It also suffers from reporting biases and often does not reflect exposure over the life course [10].

Measures of discrimination grounded in objective indicators rather than self-report typically focus on just one context, such as residential segregation or school quality [11]. Such measures are also associated with health outcomes. For example, the racial disparity in Alzheimer's disease prevalence can be partially explained by racial disparities in school quality [12]. Researchers have begun to develop measures that rely on objective indicators of structural discrimination across contexts. Dougherty and colleagues employed county-level indicators of housing and school segregation, racial disparities in high school graduation, poverty, incarceration, access to primary care, and preventable hospital admissions [13]. Lukachko and colleagues employed state-level indicators of racial differences in political participation, employment and job status, educational attainment, and incarceration/judicial treatment [14]. This work has pushed the science forward because these measures cross contexts and do not rely on self-report of discrimination. However, no validated measure of discrimination exists which assesses exposure to structural discrimination across the life course as well as multiple contexts and geographies using objective indicators [10]. Because of measurement gaps, the overall impact of discrimination on health disparities is likely underestimated [9].

Chantarat and colleagues recently published on their important work developing multidimensional measures of structural discrimination [15]. Hardeman et al [16] published a vital article on the challenge and importance of measuring structural racial discrimination and highlighted the multidimensional nature of structural discrimination. The dimensions are mutually reinforcing and not merely additive. She also highlighted the ways in which the intersections among identities such as gender, age, and disability impact the ways in which racism is experienced [16]. These studies and thought pieces push measurement scholars to move beyond a simple quantification of exposure to structural discrimination (i.e., “more exposure” vs “less exposure”) but to date have not employed a lifecourse perspective. Yet, as individuals progress through their life course, they encounter new institutions that have the potential to discriminate [17]. People are exposed to the educational system as children, for example, and encounter new institutions related to work and law enforcement as they age. A comprehensive accounting of structural racial discrimination, therefore, must capture such changes over the life course [17]. The ideal measure would accommodate adjusting for “sensitive periods” for exposure to racism (such as during pregnancy, adolescence, or young adulthood), and for how racism is experienced differently at different ages, and it would allow comparisons with self-reports of discrimination. [10].

A measurement of the cumulative and compounding effects of racial discrimination over time (or, cumulative disadvantage [18]), spanning the policies and institutions that people engage with over time [10], suggests developing an instrument relevant for older adults who have lived through many life phases [19, 20]. Although individuals in multiple racial and ethnic groups in the USA are exposed to racial discrimination, we focus in this paper on US-born Black adults for this first phase of instrument development [1, 20]. The purpose of the present study was to begin to address the field’s limitations by taking a first step at designing an instrument to quantify older Black Americans’ exposure to structural racial discrimination across settings, institutions, and across the life course.

## Methods

To design this lifecourse measure, we used a multi-phase approach grounded both in theory and in the lived experiences of older Black Americans.

## Theoretical framework

**Fig. 1:**
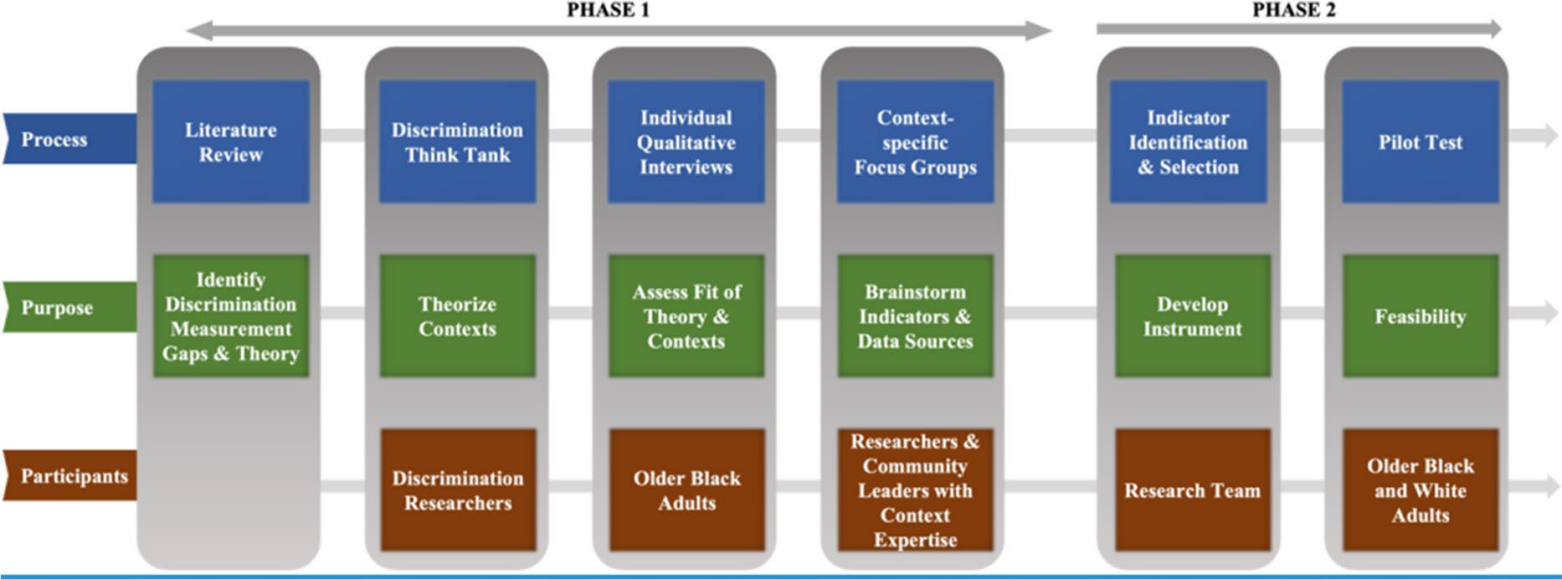
Study flow overview

We grounded the measurement in Krieger’s Ecosocial Theory on Racism and Health, which posits that people embody their exposures and circumstances: Racial disparities are physical expressions of societal inequity [20]. Exposure to injustice accumulates from utero to death, from the individual level to the global level, and through multiple “pathways of embodiment” such as economic deprivation and social trauma [20]. These injustices are neither random nor accidental; rather, power-wielding groups (e.g., white Americans and predominantly white institutions) are accountable for establishing and perpetuating them [20]. Using this theory, we sought to develop a cross-time, cross-space, and cross-context instrument (Fig. 1).

In developing our instrument, we theorized underlying contexts in which discrimination is structured and sought to identify indicators reflective of discrimination in each. This perspective treats contexts as “constructs,” and we originally thought the resulting measurement would be a scale rather than an index. [21].

### Phase 1: Foundational Qualitative Work

First, we conducted a rigorous exploratory qualitative phase. We began by convening discrimination experts from across the country for a one-day “Discrimination Think Tank.” We engaged 40 researchers in identifying and prioritizing potential contexts across which structural racial discrimination may be most salient to health outcomes. They were a combination of sociologists, nurse researchers, social epidemiologists, social workers, and physicians all working on racial health disparities or structural racial discrimination. Based on review of the literature, Krieger’s theory, and researcher input, we theorized nine contexts: civics (voting, political representation), education, employment, environment, healthcare, income/wealth and credit, media and marketing, neighborhood factors, and policing. These contexts overlapped considerably with categories previously defined or identified by other researchers and have strong face and content validity [1, 11, 13, 14, 20].

We then facilitated semi-structured individual qualitative interviews with 15 older Black Americans to add to our understanding of the contexts. Using a lifereview process, [22] we asked participants to describe each stage of their lives including who they spent time with, how they spent their days, and major events. We also specifically asked whether they felt that they or others in their life had been exposed to racism or discrimination in each of the nine contexts (such as education and health care). If yes, we asked them to provide specific examples to help us understand the types of indicators that may be important to include in the instrument. They described discrimination in each context—for example, being exposed to shortened school years, being passed over for promotions, and differential resources in redlined neighborhoods.

Although individuals are savvy, they do not always perceive the structural racism that is directed toward them, nor do they always have insights about broader patterns of inequality beyond their personal experience. To gain complementary insights from people who are familiar with how some organizations reify structural racism, we next conducted 9 virtual focus groups (one per context, such as “education”) with 38 context-specific experts (4–7 experts per group) to identify potential indicators for each context. The questions were designed to elicit measures within the context that were already available and others that could possibly be accessed in the future. Experts included health disparities researchers, a program officer from a nonprofit organization focused on environmental justice, criminal justice researchers, and a city councilperson who focuses on addressing education inequity.

During each focus group, we first asked participants to brainstorm on their own about how we could measure exposure to racial discrimination at the structural level within that context, such as the environment. Next, each person shared these ideas, and we facilitated a group dialogue to clarify and expand upon ideas, and to probe for specific examples of possible data sources or metrics. Participants who were unable to attend a focus group completed an individual interview with first author (S.E.L.). Focus groups and interviews were recorded for accuracy but were not coded for themes or transcribed verbatim. Following a focus group or individual interview, each participant individually completed a survey to identify the three most important context indicators to include.

### Phase 2: Instrument Design & Indicator Identification

To draft indicators, we first reviewed each suggestion from the focus groups to determine whether it was feasible to include in the instrument. For example, focus group participants discussed the potential value of capturing disproportionate marketing of unhealthy products to Black buyers, but we could not identify a relevant nationwide dataset in which this indicator could be assessed. If a context had more than three feasible indicators, we prioritized those that had been rated highly by focus group participants. In the absence of data on how to weight the indicators, we present three indicators per context for now to avoid making assumptions of relative contribution of each domain. For example, participants suggested dozens of potential environmental indicators but particularly emphasized the value of capturing air quality; thus, we included a composite measure of air quality. If a context had fewer than three feasible indicators, we developed indicators based on our literature review and the interviews conducted with older adults. For example, feasible focus group suggestions for civic measures were sparse, but we included political representation by race as a potentially important indicator identified by older adult interviews and the literature. Based on this work and these assumptions, we developed a total of 27 indicators across the 9 contexts. In this feasibility study, we were not yet concerned with the appropriate number of indicators per context or weighting of indicators; in future iterations, we plan to assess for the relative contribution of each context and indicator.

To connect participant data to the indicators, we created a survey that collects participants’ home addresses at multiple points in their lives, their school addresses, and their race, age, sex and financial strain. We used participants’ full street addresses to identify census tracts, counties, and congressional districts to allow for linkage to objective data sources. To identify each participant’s Federal Information Processing Standards (FIPS) census tract, county ID, and state ID, we used the Federal Financial Institutions Examination Council (FFIEC) geocoding system [23]. To identify each participant’s congressional district, we used the United States House of Representatives website [24]. The information from linked objective data sets (e.g., air quality in the person’s community) became the instrument indicators, rather than the participant-facing survey items. Our goal was to create a low-burden instrument for participants that can easily be embedded into national studies.

## Findings

**Fig. 2:**
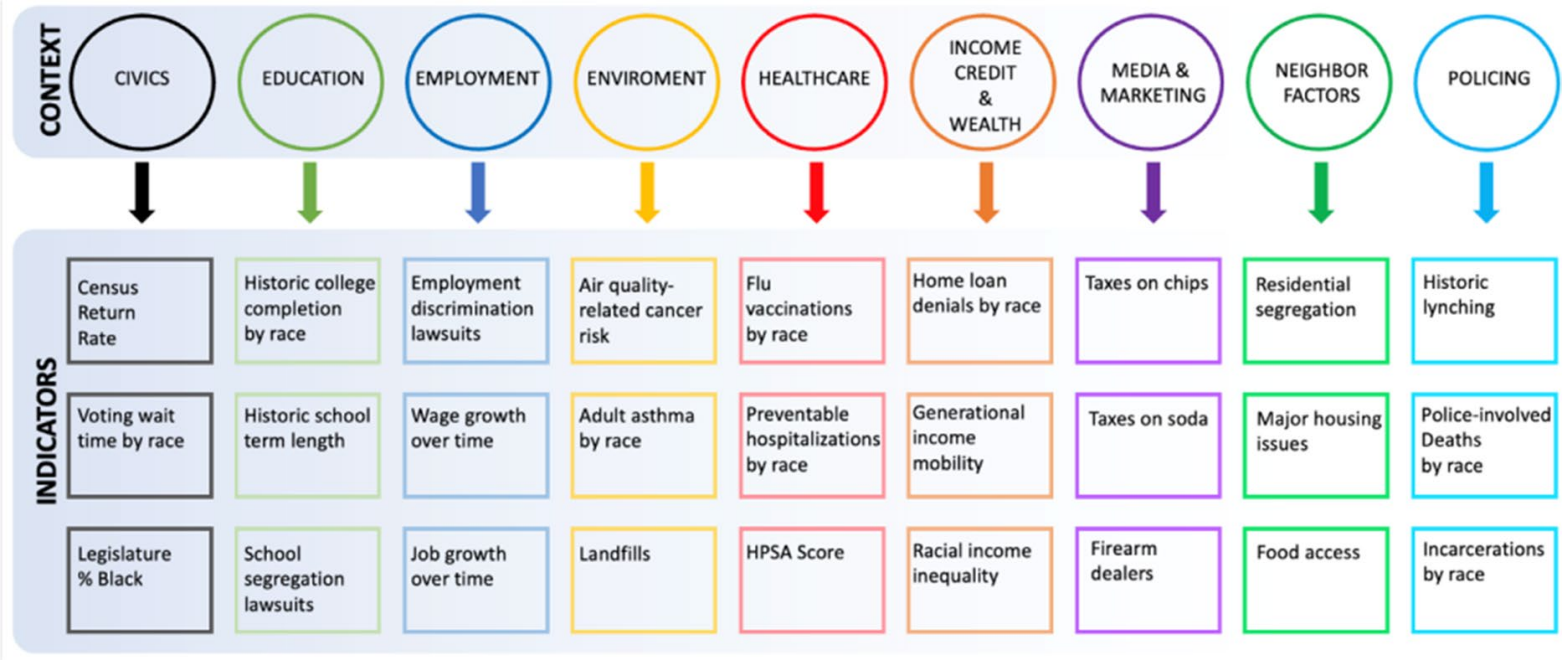
Summary of potential indicators for each context

We developed three indicators for each of the nine contexts. The 27 initial indicators are summarized in Fig. 2. Civics indicators included the census return rate in the person’s census tract, differences in voting wait times by race in their congressional district, and percent of current state legislature that is Black relative to the percentage of Black individuals in the State. Education indicators included college completion rate by race in the person’s childhood state, school term length in the childhood state by race, and school segregation lawsuits in the childhood county. Employment indicators included employment discrimination lawsuits in the state, wage growth over time in the census tract, and job growth over time in the census tract. Wage growth and job growth are examples of indicators that are not explicitly race-specific but that may represent inequitable development and distribution of employment resources based on the racial composition of neighborhoods. Environmental indicators included air-quality-related cancer risk in the census tract (a composite measure of air quality metrics publicized by the EPA), landfills in the zip code, and the racial disparity in adult asthma rate in the state. Healthcare indicators included the racial disparity in flu vaccination rate by race in the county, the racial disparity in preventable hospitalizations by race in the county, and the health professional shortage area (HPSA) score in the census tract. Within the income, credit, and wealth context, indicators included home loan denial rate by race in the county, generational income mobility in the census tract, and racial income inequality in the county. Exposure to unhealthy products and advertising was identified by focus group participants as an important concept, but we did not find any nationwide data sets on the types of media and marketing indicators that we had identified as potentially important through our foundational qualitative work (e.g., hyper-local content of billboards, radio and television media, news stories, and social media posts). We used junk food (soda and chip) taxation rates in stores and vending machines in the county and presence of firearm dealers in the zip code as proxies. For neighborhoods, we used residential segregation rates (index of dissimilarity) in the county, percentage of people in the census tract with major housing problems, and food insecurity (percentage of population in the census tract that is low income and beyond 1/2 mile from a grocery store). For policing, we used historic lynchings in the childhood county, rates of police-involved deaths by race in the state, and incarceration rates by race in the county.

“Indicators” are the actual data elements taken from publicly available data sets. “Survey items” are the pieces of information required of participants to use that indicator.

The indicators were at census tract, zip code, congressional district, county or state levels. For items that measured at the census tract or zip code level, we selected indicators that could reveal exposure to residential segregation and racist disinvestment in Black neighborhoods. For example, we used data on the percent of residents in the census tract who lack reliable access to food (neighborhood context), the generational income mobility rate in the census tract (income, credit, and wealth context), and cancer risk based on a composite measure of air quality in the census tract (environment context). For indicators that were available at broader geographic granularity such as congressional district, county, or state, we selected indicators that specifically quantified a racial difference. For example, we used data on the difference in voting wait times for Black and white voters within a congressional district (civics context), the number of federal employment discrimination charges specific to race in a county per 100,000 county residents (employment context), and the difference in preventable hospitalizations for Black and white Medicare enrollees in a county (healthcare context).

Based on the available data sets, we were able to utilize each participant’s childhood address and late adulthood address. We were not able to use young/middle-adulthood addresses or school names/addresses based on the types of data available in data sets. We were only able to identify historic data for the three indicators within the education context (all were available starting in the 1950s or earlier) and for the number of lynchings in the childhood county (which contained from the 1800s onward).

Each database required different types of data manipulation, which we completed in Excel or in Stata IC version 16. For example, for school term length, we identified the difference between the national average school term length and the school term length for Black children in the participant’s state of residence when the participant was five years old. As another example, the home mortgage loan database listed the outcome of every available home mortgage application nationwide in 2017. We removed all records that were not for an initial home purchase (e.g., refinancing), were not for an owner-occupied home, were not for a first-lien home, or for which the primary applicant was of a race/ethnicity other than non-Hispanic Black or non-Hispanic white. Although redlining and racism in the banking industry can impact all types of lending, in this feasibility study we focused on owner-occupied initial purchases to reduce heterogeneity in the data. We then calculated the number of denials by race in a county, pulled the population by race of that county from a separate census data base, calculated the Black loan denial rate and white loan denial rate, calculated the difference between the two, and linked that number to the participant’s data using county ID.

After deciding on these indicators for an initial feasibility study, we piloted the process of linking participant home addresses with the posited 27 indicators in a sample of 225 older adults (65% Black, 35% white) via an online survey. The participants were recruited using Qualtrics recruiting service sampling US-born, non-Hispanic Black and White aged 50 and older. To recruit participants, Qualtrics sent the survey out to multiple research panels and set a prescreening criterion that had to be met before the participants could complete the survey. Respondents had to be born in the USA, non-Hispanic Black and White, and had to be 50 and older. Twenty participants were also recruited through a small convenience community sample, which allowed us to assess whether Qualtrics-recruited responses differed in important ways from responses provided by people whose identities we could verify. The groups’ responses did not differ in important ways so we included both in our analysis.

Some participants were unable to provide a complete address history so we could not link their records to all indicators. For example, if a person remembered their childhood state but not their childhood address or county, we could calculate historic school term length (which is state-based) for that person but not historic lynchings in their county. In some cases, public datasets were missing data for a participant’s census tract, county, or state. After accounting for both types of incomplete records, we were left with 159 fully complete records (72%) and 220 (98%) records with at least one complete and usable address. This small feasibility pilot suggests that the general approach of linking lifecourse addresses to objective indicators from national datasets may be a feasible approach.

## Discussion

To our knowledge, this is the first study that presents a framework for assessing structural racial discrimination across contexts, geography, and the life course that is grounded in theory and in the lived experience of intended participants. Leading researchers in the field have called for improved measures of structural racism and discrimination [1, 11] and specifically for a lifecourse approach to measurement [6, 17]. This study is a step in that direction.

In this study, we developed an approach to measuring structural racial discrimination that uses a survey of lifecourse addresses and can match those addresses to available historical administrative data sets. This approach will lead to measurement of individual exposure to structural racial discrimination. This tool can be embedded into large national surveys that already measure health outcomes and become a valuable tool for understanding the association between structural racial discrimination and health outcomes such as dementia, cancer, heart disease, and disability. It could also be used to examine other non-health outcomes such as wages and incarceration. The general structure of the participant-facing survey and methods used to link to data sets to those surveys is feasible. We found datasets for many of the contextual variables we sought to match with the lifecourse address profiles. Participant-provided responses can be linked to limitless combinations of indicators without burdening participants with further questions, making reiteration, multiple instrument versions, and subsequent validity and reliability testing and algorithm generation of a low-burden process. This is the main innovation of this work.

We sought indicators that might measure structural racism over the entire life course, which represented a diversity of contexts, and could be measured with accessible data. This is a difficult task that required the use of proxy measures in several instances. For example, the indicators of media and marketing discrimination did not capture the effect of historically racist images and marketing. Other indicators, such as employment discrimination lawsuits, could indicate equity activism as opposed to the underlying segregation and discrimination we intended to assess. Others may prove too distal from health or not varied enough within a sample to include in an ultimate instrument. Based on theoretical strength and preliminary data exploration, we believe that strongest indicators identified to date include voting wait times, political representation, historic college completion rates, historic school term length, wage growth, job growth, cancer risk based on air quality, indicators of healthcare quality including but not limited to vaccination and preventable hospitalization rates, indicators of healthcare access including but not limited to HPSA score, home loan denials, generational income mobility, income inequality, residential segregation, housing issues, food access, lynchings, police-involved deaths, and incarceration rates. The policing context variables are theoretically strong and comprise useful data across multiple time periods (historic lynchings, current incarceration rates, contemporary police-involved death rates), contributing to a lifecourse perspective.

Much work is needed to address limitations of the present work. The instrument will need to be reiterated by identifying stronger indicators in some domains, identifying additional historical datasets to allow for a more extensive lifecourse measurement, and testing feasibility and eventually validity and reliability in a large sample. In particular, the indicators must include data that represent both the spatial and temporal coordinates of residence across the life course. Due to the inherent relationship between race, racism, discrimination, segregation, and socioeconomic status, assessing differences in instrument score within higher and lower SES African-Americans and Whites will be an important future test of the refined instrument’s validity [2]. Besides the measurement limitations, the proposed procedure is also limited initially disregarding other intersections of difference and discrimination such as by gender or disability. Also, in this first iteration, we examine only non-Hispanic Black and non-Hispanic White differences and ignore multiracial populations, Hispanic, Asian American, Native American and other important groups who have been discriminated against. Much of historic racial discrimination treated people as though there were only two categories of people. In our next phase, we are doing formative work with people of a wide variety of ethnicities and identities.

We had originally set forth to create a scale, under the assumption of a broader multidimensional latent construct of “structural racism.” However, this did not feel satisfactory upon further reflection sparked by exploratory analyses, because the relationships among the indicators are far more complex than we initially envisioned. One issue is that there may be multiple relevant schema for anchoring latent dimensions. We initially conceptualized “domains” as the anchoring dimensions, but it may be that timing of exposure in the lifespan or areal unit at which the exposure operates structure experience and hence indicator interrelationships as or more strongly. As a second issue, most scales traditionally posit that a latent construct (i.e., racism) manifests in numerous indicators (e.g., segregation, credit ratings, job availability). There are some conceptual and methodological considerations regarding whether the errors of these indicators are correlated with one another or not, but fundamentally, these indicators are not presumed to have causal influences on one another. However, our vision is that structural racism does manifest in various indicators, but also, that these indicators are dynamically, causally, and reciprocally related to one another [1, 25]. For example, residential segregation, police violence, and media images are not simply independent manifestations of structural racism, but also mutually cause, and reinforce, each other in complex and reciprocal ways. Media images depicting people of color as violent may help reinforce land values that shape segregation and encourage the excessive use of force by police; simultaneously, the excessive use of force by police may be interpreted by some people as justified and reinforce images of people of color as violent, which may also contribute to segregation, and so forth. Neither a latent construct hypothesized to manifest in largely independent indicators (conditional on the latent state), nor creating an index of the indicators, satisfactorily gets at these ideas that recognizes racism as a mutually reinforcing *system* of oppression, rather than simply a collection of discriminatory actions. Given this recognition, our next steps are to consider methods developed in systems science and other fields to more accurately model the complexity of these relationships between social institutions across the life course [25].

Despite limitations, this feasibility study makes important contributions to the field and is primed for iterative strengthening. We intentionally included detail about the indicators in this narrative as an invitation for collaboration with other researchers to identify improved indicators, to advocate for improved publicly available datasets if needed, and to ultimately improve measurement of structural racial discrimination through development of one or more new instruments.

There are moral and economic imperatives to address structural discrimination in the USA. Measuring structural discrimination comprehensively is crucial to the ability to make informed decisions about policies and programs intended to create racial equity. Using a new measure, policy makers, funders, and practitioners could evaluate whether a program effectively reaches not just a racially diverse population but the communities and individuals who have been most affected by historical and contemporary discriminatory policies and practices. The imperative is economic as well as moral: The Federal Reserve Bank estimates that economic cost of persistent inequities over the past thirty years adds up to $70.8 trillion in lost output since 1990 [26]. Measurement will always be inexact; striving for an inclusive, multidimensional measure is worth developing. Measurement precedes change.
